# Our knowledge about atrial fibrillation steadily increases

**DOI:** 10.1093/ehjcvp/pvaf050

**Published:** 2025-08-12

**Authors:** Stefan Agewall

**Affiliations:** Institute of Clinical Sciences, Karolinska Institute of Danderyd, Stockholm, Sweden

**Figure:**
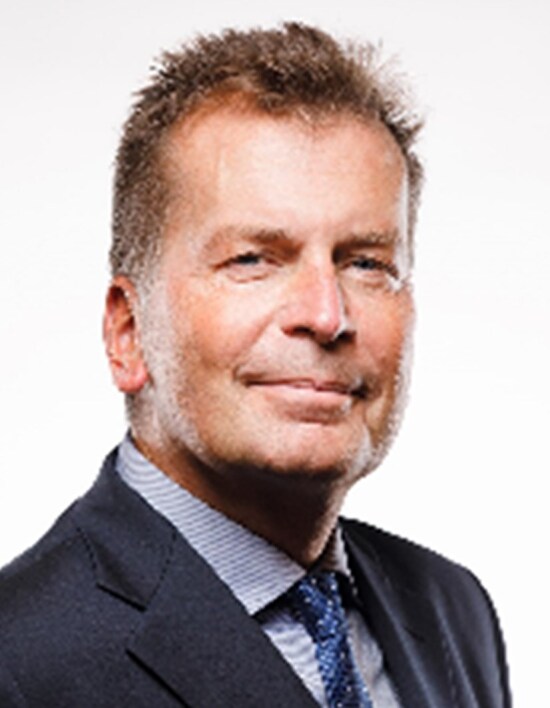


Oral anticoagulants (OACs), particularly direct oral anticoagulants (DOACs), are recommended for stroke prevention in atrial fibrillation (AF) patients. In the pivotal ARISTOTLE trial, apixaban at the standard dose (5 mg twice daily) was compared with warfarin in patients with AF, while a reduced dose of apixaban (2.5 mg twice daily) was reserved for those meeting at least two of the following criteria: age ≥80 years, body weight ≤60 kg, or serum creatinine ≥1.5 mg/dL.^1-3^ Choi *et al*.^4^ from Korea evaluated the effectiveness and safety of off-label reduced-dose apixaban vs. the on-label dose in AF patients meeting a single dose reduction criterion in a prospective cohort of 1944 Korean patients. The authors concluded that in Korean patients with AF meeting a single-dose reduction criterion of apixaban, off-label reduced-dose apixaban showed no significant differences in stroke/systemic embolism and major bleeding compared with the on-label standard dose.

Sacubitril/valsartan, an emerging medication classified as an angiotensin receptor neprilysin inhibitor, has been demonstrated to reduce major adverse cardiovascular events among patients with HFrEF, as endorsed by the American Heart Association and the European Society of Cardiology (ESC).^5-8^ A previous study has suggested a significant reduction in the risk of overall neurocognitive outcomes following use of sacubitril/valsartan compared with angiotensin-converting enzyme inhibitors (ACEIs)/angiotensin receptor blockers (ARBs).^9^ Shin *et al*.^10^ from Korea wanted to evaluate the risk of incident dementia associated with sacubitril/valsartan in patients with heart failure. They used a database consisting of 7085 users of sacubitril/valsartan and 359 153 users of angiotensin-converting enzyme inhibitors (ACEIs)/angiotensin receptor blockers (ARBs). Sacubitril/valsartan showed a 16% lower risk of dementia compared with ACEI/ARB. However, of 1180 cases of incident dementia, 1079 (91.4%) were categorized as Alzheimer's dementia and statistical significance was not reached in this main group.

DOAC binds to serum albumin at a certain level in the bloodstream and transitions between bound and unbound forms according to the dissociation equilibrium constant.^11^ With this background, Sotomi *et al*.^12^ from Japan, aimed to assess the association between albumin levels and bleeding risk in AF patients treated with DOACs. The authors used a registry of 7512 patients with AF treated with DOACs and found that a lower albumin level was independently associated with a higher bleeding risk in AF patients using DOACs. Thus, careful attention should be paid to hypoalbuminemia in the clinic when prescribing DOACs.

Whether the adoption of CHA2DS2-VA score, the sex-independent version of the CHA2DS2-VASc score is beneficial for stratifying risk of stroke in patients with AF remains unclear.^13-15^ Lip *et al*.^16^ from UK, used the data from the global, multicenter, and prospective GLORIA-AF registry. The authors compared the performances of CHA2DS2-VA and CHA2DS2-VASc in stratifying the risk of ischaemic stroke and thromboembolism (TE) and compared the risk of ischaemic stroke and TE, and second, the use of oral anticoagulants between male and female patients with AF in a total of 21 260 AF patients. The study group concluded that CHA2DS2-VA score had similar predictive performance for thromboembolic events compared to CHA2DS2-VASc score. A lower likelihood of receiving OAC among younger female patients was observed.

Sodium-glucose cotransporter-2 inhibitors (SGLT-2is) have a direct cardiac effect that is likely to be independent of its glucose lowering renal effect.^17,18^ The ability of sodium-glucose cotransporter-2 (SGLT2) inhibitors to prevent AF has been evaluated in various studies with conflicting results.^19^ Dr Pizzi and co-workers from Italy performed a trial-level meta-analysis including 52 randomized clinical studies (RCTs) (112 031 patients) comparing SGLT2 inhibitors with placebo. The authors found that SGLT2 inhibitors decreased AF risk. In the subgroup analysis, SGLT2 inhibitors were influenced by HF. Their protective effect was confirmed in the HFrEF subgroup, but not in RCTs recruiting patients with HFmrEF/HFpEF.

Rubboli *et al*.^20^ from Italy and the UK present a current opinion paper, regarding the treatment of patients with chronic coronary syndrome on long-term antithrombotic therapy who develop AF or venous thromboembolism and therefore an indication for OAC.

Therapeutic advances have significantly improved the survival of patients with cancer. Given the extension of lifespan and the rapid growth of anticancer drugs indications, these novel therapies are associated with a concomitant increase in the prevalence of adverse drug reactions (ADRs), including cardiovascular ADRs.^21,22^ Legallois *et al*. from France used the World Health Organization's VigiBase® individual case safety report database, with the aim to establish the association between anticancer drugs and cancer therapy-related cardiac dysfunction (CTRCD) reporting. Among 36 580 288 database reports, 42 828 CTRCD cases associated with at least one anticancer drug were identified with death reported in 20.6% of cases. The authors identified 25 anticancer drugs significantly associated with CTRCD reporting. It highlights discrepancies compared to drugs recommended for cardiac dysfunction evaluation in the 2022 ESC Guidelines.^21^ This underscores the importance of including CTRCD as a safety endpoint in anticancer studies.

Hypertrophic cardiomyopathy (HCM) is the most common inherited heart disease and associated left ventricular outflow tract (LVOT) obstruction can result in symptoms (e.g. dyspnoea, angina, or syncope) and reduced functional capacity.^23,24^ Clinical trials or observational studies that assessed the changes associated with beta-blockers (BBs), calcium channel blockers (CCBs), disopyramide, or cardiac myosin inhibitors (CMIs) in LVOT gradient at rest or with provocation in 1 898 patients with obstructive HCM were included in a meta-analysis by Awad *et al*.^25^ from the USA. The study showed that the different pharmacological therapies effectively reduced LVOT gradients in obstructive HCM patients to varying degrees, with disopyramide and CMIs showing the highest effect, followed by BBs and CCBs.

Enjoy the journal and the summer (northern hemisphere).
